# Increasing Obesity Rates Worldwide from 1976 to 2016: The Obesity Epidemic

**DOI:** 10.3390/jcm15010394

**Published:** 2026-01-05

**Authors:** Karsten Keller, Volker H. Schmitt, Omar Hahad, Christine Espinola-Klein, Lukas Hobohm

**Affiliations:** 1 Department of Cardiology, University Medical Center of the Johannes Gutenberg-University Mainz, 55131 Mainz, Germany; volker.schmitt@unimedizin-mainz.de (V.H.S.); omar.hahad@unimedizin-mainz.de (O.H.); espinola@uni-mainz.de (C.E.-K.); lukas.hobohm@unimedizin-mainz.de (L.H.); 2 Center for Thrombosis and Hemostasis (CTH), University Medical Center of the Johannes Gutenberg-University Mainz, 55131 Mainz, Germany; 3 German Center for Cardiovascular Research (DZHK), Partner Site Rhine Main, 55131 Mainz, Germany

**Keywords:** obesity, prevalence, epidemiology, epidemic, sex-specific

## Abstract

**Background:** Obesity is a major health concern worldwide and the World Health Organization (WHO) has declared it a global epidemic. We aimed to analyze temporal trends of obesity prevalence worldwide. **Methods:** We used data of “The Global Health Observatory” of the WHO and analyzed data from the Global Burden of Disease (GBD) Study 2023. Obesity prevalence (crude estimates) among adults in different worldwide WHO regions and temporal trends from 1976 and 2016 were analyzed. **Results:** Obesity prevalence showed large regional differences. In 2016, obesity prevalence was highest in the WHO European region and the region of the Americas, at more than 20%, whereas prevalence was lower in the WHO African region, the WHO Western Pacific region and the WHO South-East Asia region, at less than 10%. The absolute increase from 1976 to 2016 comprised an increase of 19.7% in the region of the Americas, of 14.8% and 14.2% in the WHO European region and the WHO Eastern Mediterranean region, followed by 7.3% in the WHO African region, 6.0% in the WHO Western Pacific region, and 4.2% in the WHO South-East Asia region. We observed a substantially higher prevalence of obesity in females. High BMI has risen sharply in rank worldwide, now ranging among the top six global risk factors for death. Major BMI-related causes include ischemic heart disease, type 2 diabetes mellitus, hypertensive heart disease, and ischemic stroke. **Conclusions:** Obesity prevalence showed large regional differences and was highest in Europe and America. The prevalence of obesity increased worldwide between 1976 and 2016. Obesity prevalence was higher in females than in males. The importance of obesity for premature death increased between 1990 and 2023.

## 1. Introduction

Obesity is an increasing problem worldwide, and the World Health Organization (WHO) has declared it a global epidemic [1,2]. During the past decades, an increase in overweight and obesity was recorded worldwide and in most countries [1,2,3,4,5]. Epidemiologic studies revealed that the prevalence of obesity has doubled in most countries worldwide since 1980, rising not only in high-income countries, but also in those deemed to be low-income and middle-income nations [1,2,3]. It has been estimated that more than half of the American adult population will be obese in 2030 [6]. In particular, the morbid obesity with body mass index (BMI) > 40 kg/m^2^ is a major health concern [7].

Obesity is associated with the development of a wide range of acute and chronic secondary diseases such as coronary artery disease, myocardial infarction, stroke, and renal and neurological diseases [5,8,9,10,11,12,13,14]. Obesity and chronic diseases emerged to be leading health concerns in the present and last century [15]. During the past century, growing prosperity and better living conditions resulted in lifestyle changes with higher per capita food supplies, increasing consumption due to cheaper and larger high-calorie meals, and sedentary lifestyle with decreasing physical activities that favour a positive energy balance accompanied by weight gain [15,16,17]. Since obesity is the main consequence of sedentary unhealthy lifestyle and—as aforementioned—associated with secondary acute and chronic diseases, it is strongly involved in rising epidemics of chronic disease [15,16]. Obesity can lead to insulin resistance, arterial hypertension, and dyslipidaemia, and is related with type 2 diabetes mellitus, cardiovascular disease, and non-alcoholic fatty liver disease resulting in reduced life expectancy [16,18,19]. It was estimated that overweight and obesity was related to 3.4 million deaths worldwide in the year 2010 [3]. Studies emphasized that obesity is responsible for 3.9% of years of life lost and 3.8% of disability-adjusted life-years worldwide [3]. The NCD Risk Factor Collaboration (NCD-RisC) published a pooled analysis of data from 3663 population-based studies with 222 million participants reporting an increasing age-standardized adult prevalence of obesity in 188 countries (94%) for women and in all except one country for men with a posterior probability of at least 0.80 from 1990 to 2022 [20]. Another project of the NCD-RisCV group analyzing data of 2009 population-based studies showed that the increase in obesity was regionally different with more than 55% of the global rise in mean BMI from 1985 to 2017 was driven by an increases in BMI in rural areas [21]. The Global Burden of Disease Study (GBD) revealed that the worldwide health burden attributable to high BMI has grown significantly between the years 1990 and 2021 [22]. In this observational period, the global deaths and DALYs attributable to high BMI increased more than 2.5-fold for females and males [22]. In this context, it has to be mentioned that the WHO definition of obesity (BMI ≥ 30 kg/m^2^) and the GBD concept of “high BMI” (BMI ≥ 25 kg/m^2^) are not congruent [1,23]. Although these studies provide insights on the regional change in the prevalence of obesity, the WHO data might help to detect temporal trends of obesity not influenced by inclusion criteria of large studies [23]. The WHO supports member states to strengthen their capacity to collect, compile, manage, analyze and use health data mainly derived from population-based source [23]. Thus, our objective was to investigate the increase regarding the prevalence of obesity in the different regions of the world by unaffected WHO data.

## 2. Materials and Methods

This is an epidemiological, widely descriptive study that uses aggregated data from the WHO and GBD datasets. For the present analysis, we used the data from the World Health Organization’s (WHO) “Global Health Observatory”(observational period: 1976–2016; date of access: 12 November 2024), with underlying data provided by WHO and countries from the different WHO regions [23,24]. WHO data comprise gathered health statistics (with data about mortality, morbidity, and life expectancy) from national vital records, surveys, and health facilities. These summarized data about obesity are produced using data from multiple consolidated sources, including national vital registration data, latest estimates from WHO technical programmes, United Nations partners and inter-agency groups, as well as the Global Burden of Disease and other scientific studies. After gathering the data, these were synthesized via robust scientific and statistical analysis in order to provide comparable global, regional, and country-level estimates [23,24]. For our present study, we assessed the regional prevalence (crude estimates) as proportion (in %) of obesity among adults (≥18 years) in the different WHO regions of the world at the time points 1976, 1986, 1996, 2006, and 2016. The definition of the WHO regions and the belonging countries is provided in the Supplementary Material [25]. These time points were used to illustrate the substantial increase in obesity prevalence. BMI ≥ 30 kg/m^2^ was used as the definition of obesity (Table 1) [23,26]. Regional data with provided confidence intervals from the WHO’s “The Global Health Observatory” were used [23]. Confidence intervals were directly obtained from data sources.

Additionally, we obtained and analyzed data from the Global Burden of Disease (GBD) Study 2023, accessible via the Institute for Health Metrics and Evaluation (IHME) (https://vizhub.healthdata.org/gbd-compare/ (accessed on 1 April 2025)) [22]. The GBD provides harmonized estimates of mortality, morbidity, and risk factors for all countries and world regions based on a comprehensive synthesis of vital registration systems, epidemiological surveys, and administrative health data [22]. Our study utilized data from the GBD Study 2019 with a comprehensive assessment encompassing epidemiological variables such as incidence, prevalence, deaths, YLDs, YLLs, and DALYs across 369 diseases and injuries, 286 mortality causes, and 87 risk factors in 204 countries and regions [27]. For this analysis, we extracted age-standardized estimates for mortality attributable to high body mass index (BMI ≥ 25 kg/m^2^) across global and regional levels, stratified by sex and year (1990–2023). Data were processed descriptively to visualize temporal shifts in the ranking of risk factors and causes of death associated with elevated BMI [22]. The pertinent data on the burden of disease were sourced from the GBD Results tool on the Institute for Health Metrics and Evaluation website (https://www.healthdata.org/data-tools-practices/interactive-data-visuals (accessed on 1 April 2025)) [27].

We selected these two data sources to analyze, in trusted datasets, both the prevalence of obesity and the importance of obesity in the context and interplay of diseases of various organ systems. Although these two datasets have different definitions of increased body weight, they are the largest datasets in this scientific field, making it possible to obtain reliable results and draw conclusions from them.

### 2.1. Ethics

Since our study is based not on direct access to individual patient data but only on summarized results provided by the WHO and the GBD database, approval by an ethics committee and patients’ informed consent are not required, in accordance with German law.

### 2.2. Statistics and Illustration

We assessed the prevalence of obesity in the WHO regions at different time points comprising the years 1976, 1986, 1996, 2006, and 2016 [23]. Temporal trends in crude prevalence estimates in percent and the associated 95% confidence intervals were assessed and graphically illustrated for females, males and both sexes. Excel tools were used for graphical illustration. Temporal trends regarding obesity prevalence were estimated by means of linear regression analyses. Results were presented as β and 95% confidence intervals (CI). Linear regression was used in several similar investigations. The timepoints were chosen to analyse 10-year changes. All statistical analyses were carried out with the use of SPSS software (IBM Corp. Released 2017. IBM SPSS Statistics for Windows, Version 25.0. IBM Corp: Armonk, NY, USA). Only *p* values < 0.05 were considered to be statistically significant.

## 3. Results

**Figure 1:**
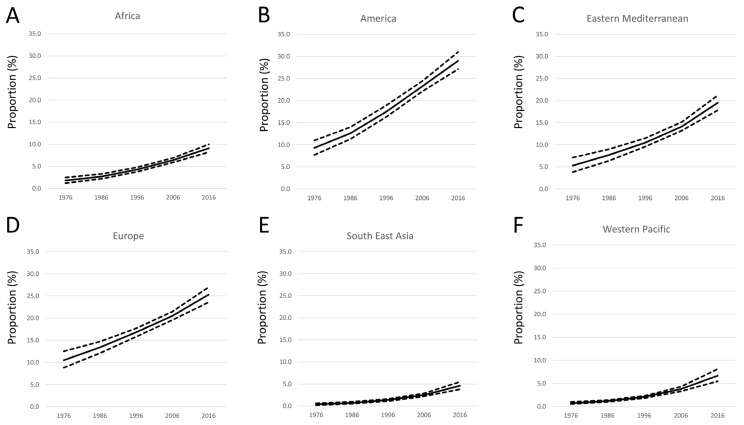
Temporal trends regarding obesity in different continents/regions ((**A**) WHO African region; (**B**) WHO region of the Americas; (**C**) WHO Eastern Mediterranean region; (**D**) WHO European region; (**E**) WHO South-East Asia region; (**F**) WHO Western Pacific region) among adults (mean (solid lines) and 95% confidence interval (dashed lines)).

This worldwide analysis of temporal trends of the obesity prevalence across different WHO regions identifies substantial regional differences and a strong time trend, with increasing obesity prevalence in all investigated regions among adult populations (Figure 1).

**Figure 2:**
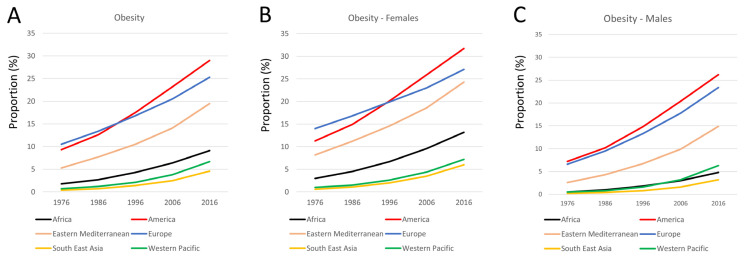
Temporal trends regarding obesity in different continents/regions among adults (mean values) regardless of sex (**A**), in females (**B**) and in males (**C**).

In the year 1976, prevalence of obesity was highest in the and the WHO region of the Americas at 10.5% and 9.3%, while the lowest rates were detected in the WHO South-East Asia region and the at 0.4% and 0.7% (Figure 1 and Figure 2). We observed a strong increase regarding the prevalence of obesity in all WHO regions, while the strongest increases were detected in the WHO Eastern Mediterranean region, the WHO European region, and the WHO region of the Americas (Figure 1 and Figure 2).

In the year 2016, the highest obesity prevalence was identified in the WHO region of the Americas at 29.0% and the WHO European region at 21.2%, followed by the WHO Eastern Mediterranean region at 19.5%. In contrast, the prevalence rate of obesity in the WHO African region, the WHO Western Pacific region and the WHO South-East Asia region was far behind the aforementioned regions with 9.1%, 5.5% and 4.6%. The absolute increase from 1976 to 2016 showed a total increase of 19.7% in the WHO region of the Americas, of 14.8% and 14.2% in the WHO European region, and the WHO Eastern Mediterranean region, followed by 7.3% in the WHO African region and 6.0% in the WHO Western Pacific region, as well as 4.2% in the WHO South-East Asia region. In contrast, the relative increase was highest in the WHO South-East Asia region and in the WHO Western Pacific region with an 11.5-fold and a 9.6-fold increase. While the relative increase in obesity prevalence was 5.1-fold in the WHO African region, the relative increase was lower in the WHO Eastern Mediterranean region and in the WHO region of the Americas—3.7-fold and 3.1-fold—and lowest in the WHO European region at 2.4-fold. The linear regressions for all regions showed a significant increase in obesity prevalence. Statistically, the largest increase was observed in the WHO South-East Asia region (β_per 10 years_ 8.80 [95%CI 3.35–14.26], *p* = 0.014), the WHO Western Pacific region (β_per 10 years_ 6.18 [95%CI 2.45–9.92], *p* = 0.013), and the WHO African region (β_per 10 years_ 5.28 [95%CI 3.44–7.11], *p* = 0.003), followed by the WHO Eastern Mediterranean region (β_per 10 years_ 2.79 [95%CI 1.92–3.67], *p* = 0.002), the WHO European region (β_per 10 years_ 2.70 [95%CI 2.22–3.18], *p* < 0.001), and the WHO region of the Americas (β_per 10 years_ 1.98 [95%CI 1.61–2.35], *p* < 0.001). We observed a substantially higher prevalence of obesity and a stronger increase regarding the prevalence of obesity in females in the WHO region of the Americas, the WHO European region, and the WHO Eastern Mediterranean region, followed by the WHO African region compared to males during the observational period between 1976 and 2016. In contrast, the prevalence of obesity was only slightly higher in females than in males in the WHO South-East Asia region and in the WHO Western Pacific region (Figure 2). The absolute increase regarding the prevalence of obesity was highest in both sexes in the WHO region of the Americas at 20.4% in females and 19.0% in males during the observational period, followed by the WHO European region with higher increase in males (16.8%) compared to females (13.1%). In the WHO Eastern Mediterranean region, the temporal trends also showed a high increase in women, at 16.1%, and a smaller increase of obesity prevalence in men, at 12.3% (Figure 2).

**Figure 3:**
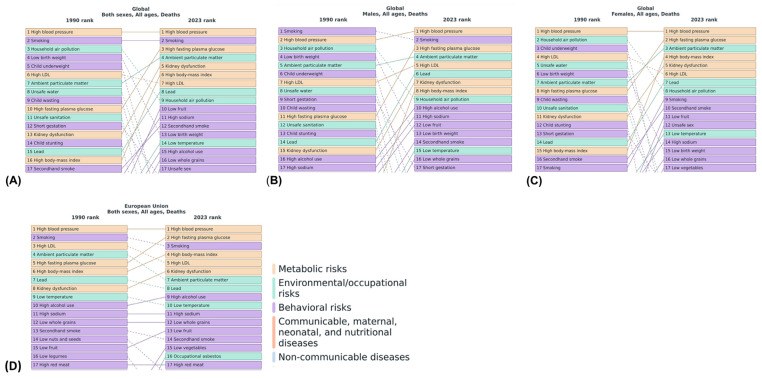
Global Burden of Disease 2023: trends in mortality risk factors and causes linked to high body mass index. Panels (**A**) to (**D**) show temporal shifts in the leading global and regional risk factors for all-cause mortality from 1990 to 2023, stratified by sex and region ((**A**), both sexes; (**B**), males; (**C**), females; (**D**), European Union). Lines between the time points help to understand the change of the places between the time points.

Regarding risk factors for mortality in the population, high blood pressure and smoking were the two most common cardiovascular risk factors for premature death worldwide in the years 1990 and 2023 (Figure 3). High BMI as a risk factor climbed from the 16th place in 1990 to the 6th place in 2023 in terms of its importance for premature death worldwide. In combination with higher rates of obesity in recent years, the high fasting plasma glucose became the third most important risk factor for mortality worldwide in the year 2023. While high BMI was not an important risk factors for mortality, ranked among the first 20 risk factors in men during the year 1990, high BMI became the 8th most important risk factor for mortality in males worldwide in the year 2023. Remarkably, the importance of high BMI as a mortality risk factor for females increased between 1990 and 2023, illustrated by its shift from the 15th to the 4th place on the list of the most important risk factors for premature death in women from the year 1990 to the year 2023 (Figure 3).

**Figure 4:**
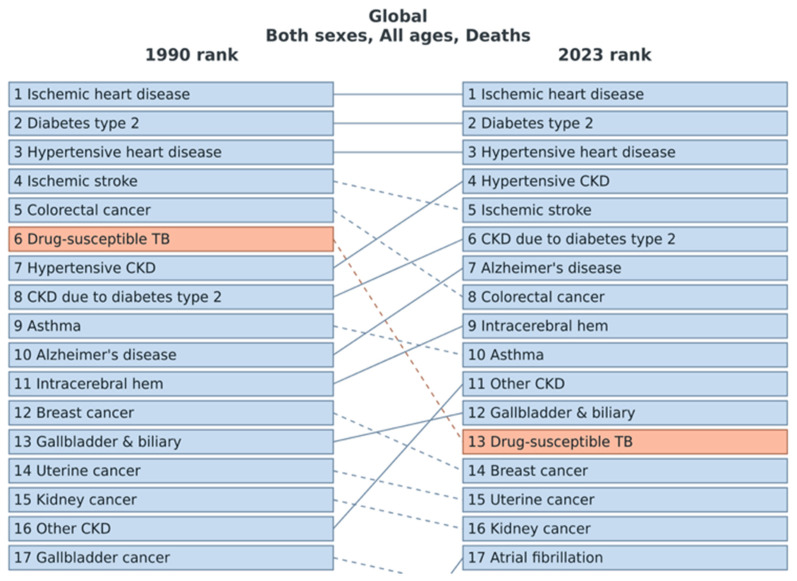
Global Burden of Disease 2023: trends in mortality risk factors and causes linked to high body mass index.

The leading causes of death linked with high BMI did not change in the first through third place from 1990 to 2023, comprising ischemic heart disease in first place, diabetes mellitus in second place, and hypertensive heart disease in third place. In contrast, hypertensive chronic kidney disease and chronic kidney disease due to diabetes mellitus increased in importance from 1990 to 2023 (Figure 4).

## 4. Discussion

The results of our study analyzing worldwide temporal trends of obesity prevalence in the adult population of different regions showed that the prevalence of obesity increased dramatically during the last four decades. Thus, it is no surprise that the WHO has declared obesity a global epidemic [1,2].

The data of the present study reveal an obesity prevalence of approximately 10% in the WHO region of the Americas and the WHO European region already in 1976. From this time point, the prevalence increased to nearby 30% in the WHO region of the Americas and approximately 20% in the WHO European region and the WHO Eastern Mediterranean region, indicating an alarmingly high prevalence in these regions during the year 2016. Temporal trends revealed a significant increase of approximately 20% in the WHO region of the Americas and 15% in the WHO European region and the WHO Eastern Mediterranean region.

The reported worldwide prevalence rate of overweight and obesity in the GBD Study was at 36.9% in adult men and 38.0% in women during the year 2013, higher than the identified prevalence in our study. In this context, it has to be mentioned that the WHO definition of obesity (BMI ≥ 30 kg/m^2^) and the GBD concept of “high BMI” (BMI ≥ 25 kg/m^2^) are not congruent; therefore, the GBD detects higher rates of the combination of overweight and obesity compared to the obesity definition in our study [1,3,23]. One other GBD study reported that the worldwide health burden attributable to high BMI has grown significantly between the years 1990 and 2021 [22]. As mentioned in the introduction, obesity prevalence and the increase in obesity prevalence show large regional differences. The NCD Risk Factor Collaboration (NCD-RisC) group published some papers about this topic, showing an increasing age-standardized adult prevalence of obesity in 188 countries (94%) for women and in all except one country for men between the years 1990 and 2022 [20]. Studies reported the highest rate of obesity in the Pacific Islands and the lowest rates in Asian countries, whereas obesity prevalence was most frequently higher in developed countries [10,12]. Notably, the increase in obesity prevalence was primarily identified in rural areas [21]. In accordance with the literature, obesity prevalence was high in the densely populated and highly developed countries of Europe and North America [12]. The situation in the USA is particularly well researched and, at the same time, particularly alarming, emphasizing that roughly one-third of the adults are obese [6,13,28]. The high prevalence of obesity in the United States is a main driver for the high prevalence of obesity in the WHO region of the Americas in our present study. For Europe it is well known that the prevalence pattern differs markedly between the European countries [5,28,29]. While the WHO data revealed an increase regarding the prevalence of obesity by 15% in the WHO European region during the observational period between 1976 and 2016, other studies emphasized an increase by approximately 30% over the past 15 years [5,30]. Although the GBD and the NCD-RisC studies provide important insights into the regional changes in obesity prevalence, WHO data might help detect temporal trends in obesity that are not influenced by the inclusion criteria of large studies [23].

In accordance with the literature, our study results indicate an increase in obesity prevalence in both sexes [13]. However, our data underline sex-specific differences in obesity prevalence [20,31,32]. In accordance with other studies [31,32,33], our results showed a higher prevalence of obesity in women. Others studies confirmed that women’s obesity prevalence exceeds men’s in the majority of countries [33]. Women face large weight management challenges driven by hormonal changes during pregnancy, perimenopause, and menopause, which not only affect fat distribution and filling of fat cells, but also increase cardiovascular–kidney–metabolic syndrome risk [34]. Current clinical guidelines often overlook sex-specific factors, impacting the effectiveness of obesity management strategies in women [34]. The gap in obesity prevalence between males and females differed substantially between the regions, and another study reported a median gap of 6% with higher prevalence in women worldwide compared to men [33]. Since obesity is a multifactorial condition affected by complex interactions among sex, sociocultural, environmental, and physiological factors, it is important to investigate the mechanisms underlying these sex-specific differences in prevalence in more detail, taking comorbidities, socioeconomic, psychological, and treatment factors into account [32].

Although the prevalence rate of obesity in children is not the primary focus of our study, the prevalence of overweight and obesity in children has significantly increased in recent years [3]. Studies underlined that in developed countries, 23.8% of the boys and 22.6% of the girls were overweight or obese in the year 2013 [3], and the prevalence of obesity in children and adolescents worldwide was reported to be around 8.5% (95% CI 8.2–8.8) [29]. This is of special interest because adolescents affected by overweight or obesity had a 70% risk to become overweight adults [13]. This risk increases to 80% if one or both parents are overweight or obese [13].

Many epidemiological studies have indicated that obesity strongly contributes to the development of a wide range of secondary diseases [6,12,13,35]. Related diseases are cardiovascular diseases [5,6,8,9,10,11,12,13,35,36], type 2 diabetes [5,6,8,12,13,35,37], cancer [5,12,13,35,38,39,40,41], liver diseases and gastroesophageal reflux [12,13,35,41], lung and sleep disorders [6,13,37,38,41,42,43,44,45], chronic kidney disease [46,47,48], arthritis [6,49,50,51], and psychiatric disorders [52,53,54,55,56,57,58,59,60]. The risk for obesity-related secondary comorbidities increased significantly with a rise in BMI [26]. Moreover, obesity is connected with significant disability [6,8,61,62], excess in mortality [6,8,9,13,38,39,61,62,63,64], lower productivity and higher medical costs [6].

Although there are some debates regarding the impact of obesity on excess mortality, it is undeniable that obesity is associated with an underestimated excess rate regarding mortality rate in the population [6,13]. Our analysis of the GBD database emphasizes that the importance of obesity for premature death in populations increased worldwide from 1990 to 2023.

The excess mortality is primarily caused by an increase in obesity-related risk factors like diabetes mellitus and arterial hypertension, which promote cardiovascular diseases such as coronary artery disease, myocardial infarction, stroke and heart failure, and several other health conditions, including asthma, cancer, degenerative joint disease, and many others [13]. Our analysis of the GBD points in the same direction, showing that ischemic heart disease, diabetes mellitus, and hypertensive heart disease are the most important causes of death linked to obesity worldwide. Therefore, obesity accounts for a heavy burden on the public and on health-care systems worldwide every year [6,12,13,35,37,65]. Beside these direct health care costs, obesity is responsible for additional costs associated with work-lost days, restricted activity and productivity, bed days and downtime during physician visits [66,67]. Approximately 2% of the direct costs of the health care system expenses in France in the year 1992 and approximately 4% of the health care expenses for direct and indirect costs in Germany in the year 2008 were related to obesity [66,68]. Therefore, obesity plays an important role of obesity as a health care factor with significant social and economic effects and should not be underestimated in the light of its increasing prevalence worldwide. In addition to changes in lifestyle, including nutrition behaviour and physical activity, obesity prevention starting in childhood is an important tool to address rising obesity rates [69]. Several lifestyle behaviours may influence body weight and determine whether a person can maintain energy balance over the long term [69]. In particular, the consumption of sweets, sugar-sweetened beverages, and processed food makes it difficult to maintain a balance between energy input and energy expenditure [69]. Physical activity also influences long-term weight gain, but evidence remains inconsistent [69]. New strategies might may include the use of biomarkers and therapeutic interventions [70,71]. The strengths of our study include the long historical window, harmonized GBD mortality estimates, and global coverage.

## 5. Limitations

There are several limitations of our study that should be mentioned. First, the use of crude prevalence instead of age-standardized data is a main limitation. Second, since we predefined the time-points of the analysis, variations in obesity prevalence might have gone undetected. Third, differing BMI definitions across the two datasets might hamper direct comparison of results. Fourth, the ecological study design, heterogeneity in data quality, and differences in surveillance capacity across regions and over time must be considered.

## 6. Conclusions

Obesity prevalence showed large regional differences and was highest in the WHO European region and the region of the Americas. Overall, obesity prevalence increased significantly worldwide during the observational period from 1976 to 2016. While the relative increase in obesity prevalence was 5.1-fold in the WHO African region, this increase was lower in the WHO Eastern Mediterranean region and in the WHO region of the Americas, with 3.7-fold and 3.1-fold increases, and lowest in the WHO European region with a 2.4-fold increase. The obesity prevalence was higher in females than in males. Therefore, obesity therapy and prevention remain major public health priorities. Strong efforts must be made to prevent further increases in obesity prevalence. The importance of obesity was also highlighted by its increasing contribution to premature death from 1990 to 2023.

## Figures and Tables

**Table 1:** Obesity categorization stratified by BMI (WHO, CDC).

Category	Body Mass Index
Underweight	<18.5 kg/m^2^
Normal weight	18.5–24.9 kg/m^2^
Overweight	25.0–29.9 kg/m^2^
Obesity Type I	30.0–34.9 kg/m^2^
Obesity Type II	35.0–39.9 kg/m^2^
Obesity Type III	≥40 kg/m^2^

## Data Availability

All data from this study are publicly available online. We used the data from the World Health Organization’s (WHO) “Global Health Observatory” (observational period: 1976–2016; date of access: 12 November 2024), with underlying data provided by WHO and countries from the different WHO regions and GBD, also publicly available online.

## Supplementary Materials

The following supporting information can be downloaded at https://www.mdpi.com/article/10.3390/jcm15010394/s1.
