# Supplementary material for: Increasing Obesity Rates Worldwide from 1976 to 2016: The Obesity Epidemic
Source: J Clin Med. 2026 Jan 5;15(1):394. doi: 10.3390/jcm15010394 (PMC12787145; doi:10.3390/jcm15010394)
Supplement: Supplementary file 1 [file jcm-15-00394-s001.zip › jcm-3992316-supplementary.pdf]

Supplementary Material

Increasing obesity rates worldwide from 1976 to 2016: the obesity epidemic

Keller et al.: Obesity

Karsten Keller, MD[1,2]; Volker H. Schmitt, MD[1,3]; Omar Hahad, PhD[1,3]; Christine Espinola-Klein, MD[1,2]; Lukas Hobohm, MD[1,2]

Affiliations:

[1] Department of Cardiology, University Medical Center of the Johannes Gutenberg-University Mainz, Mainz, Germany

[2] Center for Thrombosis and Hemostasis (CTH), University Medical Center of the Johannes Gutenberg-University Mainz, Mainz, Germany

[3] German Center for Cardiovascular Research (DZHK), Partner Site Rhine Main, Mainz, Germany

Methods

Definition of WHO regions

The WHO defines different regions of the World:[25]

The **WHO African region** comprises the following countries Algeria, Angola, Benin, Botswana, Burkina Faso, Burundi, Cabo Verde, Cameroon, Central African Republic, Chad, Comoros, Congo, Côte d'Ivoire, Democratic Republic of the Congo, Equatorial Guinea, Eritrea, Eswatini, Ethiopia, Gabon, Gambia, Ghana, Guinea, Guinea-Bissau, Kenya, Lesotho, Liberia, Madagascar, Malawi, Mali, Mauritania, Mauritius, Mozambique, Namibia, Niger, Nigeria, Rwanda, Sao Tome and Principe, Senegal,

Seychelles, Sierra Leone, South Africa, South Sudan, Togo, Uganda, United Republic of Tanzania, Zambia, and Zimbabwe.

The **WHO region of the America** includes the countries Antigua and Barbuda, Argentina, Bahamas, Barbados, Belize, Bolivia (Plurinational State of), Brazil, Canada, Chile, Colombia, Costa Rica, Cuba, Dominica, Dominican Republic, Ecuador, El Salvador, Grenada, Guatemala, Guyana, Haiti, Honduras, Jamaica, Mexico, Nicaragua, Panama, Paraguay, Peru, Puerto Rico (*Associate WHO Member State), Saint Kitts and Nevis, Saint Lucia, Saint Vincent and the Grenadines, Suriname, Trinidad and Tobago, United States of America, Uruguay, and (Bolivarian Republic of) Venezuela.

In contrast, the **WHO South-East Asia region** consists per definition of the countries Bangladesh, Bhutan, Democratic People's Republic of Korea, India, Indonesia, Maldives, Myanmar, Nepal, Sri Lanka, Thailand, and Timor-Leste.

The WHO defines the **WHO European region** with the countries Albania, Andorra, Armenia, Austria, Azerbaijan, Belarus, Belgium, Bosnia and Herzegovina, Bulgaria, Croatia, Cyprus, Czechia, Denmark, Estonia, Finland, France, Georgia, Germany, Greece, Hungary, Iceland, Ireland, Israel, Italy, Kazakhstan, Kyrgyzstan, Latvia, Lithuania, Luxembourg, Malta, Monaco, Montenegro, Netherlands, North Macedonia, Norway, Poland, Portugal, Republic of Moldova, Romania, Russian Federation, San Marino, Serbia, Slovakia, Slovenia, Spain, Sweden, Switzerland, Tajikistan, Türkiye, Turkmenistan, Ukraine, United Kingdom of Great Britain and Northern Ireland, and Uzbekistan.

In addition, the **WHO Eastern Mediterranean region** comprises Afghanistan, Bahrain, Djibouti, Egypt, Iran (Islamic Republic of), Iraq, Jordan, Kuwait, Lebanon, Libya, Morocco, Oman, Pakistan, Qatar, Saudi Arabia, Somalia, Sudan, Syrian Arab

Republic, Tunisia, United Arab Emirates, West Bank and Gaza Strip (*Non-Member area), and Yemen.

Furthermore, the **WHO Western Pacific region** includes the countries Australia, Brunei Darussalam, Cambodia, China, Cook Islands, Fiji, Japan, Kiribati, Lao People's Democratic Republic, Malaysia, Marshall Islands, Micronesia (Federated States of), Mongolia, Nauru, New Zealand, Niue, Palau, Papua New Guinea, Philippines, Republic of Korea, Samoa, Singapore, Solomon Islands, Tokelau (*Associate WHO Member State), Tonga, Tuvalu, Vanuatu, and Vietnam.[25]

References:

25. World Health Organisation W. Countries/areas by WHO region. Internet page of the WHO 2025.
